# Increased Level of Tim-3^+^PD-1^+^CD4^+^T Cells With Altered Function Might Be Associated With Lower Extremity Arteriosclerosis Obliterans

**DOI:** 10.3389/fimmu.2022.871362

**Published:** 2022-06-10

**Authors:** Liyuan Cui, Lanting Chen, Yuxin Dai, JingMin Ou, Mingke Qiu, Songcun Wang

**Affiliations:** ^1^Laboratory for Reproductive Immunology, Hospital of Obstetrics and Gynecology, Fudan University Shanghai Medical College, Shanghai, China; ^2^Department of General Surgery, Xinhua Hospital, Shanghai JiaoTong University, School of Medicine, Shanghai, China; ^3^Department of General Surgery, Shigatse People’s Hospital, Shigatse, China

**Keywords:** Tim-3, PD-1, CD4^+^T cells, lower extremity arteriosclerosis obliterans, ox-LDL

## Abstract

Lower extremity arteriosclerosis obliterans (LEASO) is a vascular disease that may result in adult limb loss worldwide. CD4^+^T cell-mediated immunity plays a significant role in LEASO. The T cell immunoglobulin and mucin domain 3 (Tim-3) and inhibitory receptor programmed cell death-1 (PD-1) are well-known immune checkpoints that play crucial roles in regulating CD4^+^T cell activation or tolerance. In this study, blood mononuclear cells were isolated from the blood samples of healthy controls and patients who were diagnosed with LEASO for the first time [stage III or IV according to the Fontaine classification system and had not received drugs (except for heparin) or surgery treatment]. We concluded the higher proportion of Tim-3^+^PD-1^+^CD4^+^T cells in human higher stage LEASO, and oxidized low-density lipoprotein increased Tim-3 and PD-1 co-expression by activating CD4^+^T cells in a dose- dependent manner. Tim-3^+^PD-1^+^CD4^+^T cells displayed a more active status and produced more anti-atherogenic cytokines compared to Tim-3^-^PD-1^-^CD4^+^T cells. Apart from the increased frequency, the altered function of Tim-3^+^PD-1^+^CD4^+^T cells was also observed in LEASO compared to those from healthy controls. These *in vitro* results indicated that Tim-3 and PD-1 might be promising early warning targets of higher stage LEASO. In addition, the blockade of Tim-3 and PD-1 signaling pathways aggravated the pro-atherogenic Th1 responses in LEASO, further suggesting that the cardiovascular safety must be a criterion considered in using immune checkpoint inhibitors to reverse T cell exhaustion during tumors and chronic viral infections.

## Introduction

Lower extremity arteriosclerosis obliterans (LEASO) is a manifestation of systemic atherosclerosis in the limbs. The growth of atherosclerotic material and secondary thrombosis cause the thickening of the intima of lower extremity arteries and the narrowing or even occlusion of the lumen and further cause a series of symptoms and signs of the affected limb, such as intermittent claudication, resting pain, ulcer necrosis, and even limb loss (1). Notable progress has been made in the treatment of LEASO, including surgical techniques, endovascular interventions, and pharmacological treatments. However, restenosis usually relapses in 1–2 years after therapy, and LEASO remains responsible for the surgical limb loss worldwide (2). Furthermore, most patients with LEASO have no apparent clinical symptoms in the early stages, which leads to a delay in treatment. Therefore, early diagnostic methods and new therapies for LEASO are required.

Although studies have focused on the endothelial dysfunction in LEASO, chronic immune stimulation plays an important role in LEASO as it is derived and developed from atherosclerosis (1, 3, 4). In the plaque, low-density lipoprotein cholesterol is modified by oxidative processes [oxidized low-density lipoprotein (ox-LDL)] and taken up by macrophages. The intracellular lipid droplets form into foam cells as the continuous intracellular accumulation of lipids eventually exceeds the cholesterol storage capacity of macrophage. Foam cell formation and the per se pro-inflammatory properties of ox-LDL initiate immune responses with an increasing secretion of pro-inflammatory cytokines (5). In addition, a variety of cell types, including B and T cells, accumulate within the plaque and in the surrounding adventitia. The continuous secretion of inflammatory mediators is understood as a self-amplifying inflammatory cascade that ultimately promotes an unstable plaque phenotype, plaque erosion and rupture, and the formation of occlusive arterial thrombi that restrict blood flow and cause critical tissue ischemia (5, 6).

In single-cell RNA-sequencing and mass cytometry by the time of flight of human atherosclerotic plaques, T cells outnumber other hematopoietic lineages and reach a frequency of up to 65% of all leukocytes (7–9). Meanwhile, CD4^+^T cells are the predominant T cell type in mouse atherosclerotic lesions (10). It has been reported that Th1-type cytokines, IFN-γ and TNF-α, are pro-atherogenic and Th2- and Treg-type cytokines, IL-10 and TGF-β1, are anti-atherogenic, while Th17-type cytokines, IL-17A and IL-22, have controversial roles in atherosclerosis (11). However, the complex regulatory network responsible for the CD4^+^T immune responses leading to the development of atherosclerosis, and the subsequent cardiovascular diseases, especially LEASO, remains insufficiently understood.

After antigen recognition, a second signal from co-stimulatory molecules is required for T cell activation. Co-stimulatory molecules and co-inhibitory molecules, the predominant members of the immune checkpoint protein family, may either promote or hamper T cell activation and play a pivotal role in atherosclerosis (12). Studies have shown that single programmed cell death protein 1(PD-1) (13), T cell immunoglobulin and mucin domain 3 (Tim-3) (14), or cytotoxic T-lymphocyte antigen-4 (CTLA-4) (15) pathway signaling has anti-atherogenic effects during the progress of murine atherosclerosis. In addition, the co-expression of these immune checkpoints on T cells identifies T cell subsets with a unique phenotype that could shape the inflammatory responses during many diseases. The blockade of these immune checkpoints to improve T cell responses is considered as a novel strategy for the treatment of chronic infections and some tumors (16, 17).

The functional regulation of PD-1, CTLA-4, and Tim-3 on CD4^+^ T cells in human LEASO (especially the higher stage according to the Fontaine classification system) has not been previously explored. We hypothesized that the co-expression of these immune checkpoints would demarcate particularly the CD4^+^ T cell subsets that are associated with LEASO. To test this hypothesis, we investigated Tim-3, PD-1, and CTLA-4 expression on CD4^+^T cells in the context of LEASO and found a higher number of Tim-3^+^PD-1^+^CD4^+^T cells in LEASO. In particular, we also used detailed surface and intracellular phenotype analyses as well as multifunctional assays to study the role of their signaling pathways in regulating CD4^+^T cell function in LEASO.

## Materials and Methods

### Human Samples

The study enrolled 36 healthy people [healthy controls (HCs), age-, sex-, body mass index (BMI)–, blood pressure–, and fasting blood glucose–matched; **Table 1**] and 70 patients diagnosed with LEASO (stage III or IV according to the Fontaine classification system) from Xinhua Hospital affiliated with Shanghai Jiaotong University School of Medicine. Ankle-brachial index measurements were performed before the initiation of the study. B-mode ultrasonography was used in the evaluation of all the healthy volunteers and patients with (plaque in the femoral artery) or without (no plaque in artery) atherosclerosis. LEASO was angiographically documented in all patients.

**Table 1:** Clinical characteristics of enrolled subjects.

Subjects	LEASO (n = 70)	HCs (n = 36)	P-value
Age mean (years)	68.91 ± 2.53	65.20 ± 2.14	0.28
Age range (years)	55-81	58-79	–
Men/women	37/33	19/17	–
Treatment history	–	–	–
Cho (mmol/L)^a^	3.95 ± 0.34	4.38 ± 0.26	0.33
TG (mmol/L)^a^	1.43 ± 0.17	1.73 ± 0.37	0.45
HDL-C (mmol/L)^a^	1.02 ± 0.07	1.20 ± 0.12	0.20
LDL-C (mmol/L)^a^	2.40 ± 0.35	2.64 ± 0.32	0.63
FBG (mmol/L)^a^	5.82 ± 0.49	5.33 ± 0.24	0.38
SBP (mmHg)^a^	137.7 ± 4.49	135.3 ± 7.34	0.77
DBP (mmHg)^a^ BMI (kg/m^2^)^a^	81.45 ± 2.31 26.23 ± 0.92	80.20 ± 3.27 25.14 ± 0.96	0.75 0.42

LEASO, patients with lower extremity arteriosclerosis obliterans; HCs, healthy controls; Cho, cholesterol; TG, triglyceride; HDL-C, high-density lipoprotein cholesterol; low-density lipoprotein cholesterol; FBG, fasting blood glucose; SBP, systolic blood pressure; DBP, diastolic blood pressure; BMI, body mass index.

a Median ± SEM.

The diagnosis of LEASO was made by history and physical examination. All LEASO patients had the characteristic complaints of persistent pain at rest or ulcer and gangrene and confirmed by B-mode ultrasonography and angiography. All patients were diagnosed with LEASO for the first time and had not received drugs (except for heparin) or surgery treatment before sampling. Moreover, patients who were critically ill within the last 6 months were also excluded, such as cancer, serious infections, renal failure, connective tissue diseases, and hormone replacement therapy. Individuals without any of the above symptoms and other diseases (except for the varicose veins of lower limbs) were enrolled as HCs.

Femoral artery blood and elbow venous blood were collected after angiography. The patients then went through percutaneous transluminal angioplasty or cutting balloon angioplasty treatment arms based on their occlusion. All the patients and eighteen of the healthy volunteers have signed the agreements to collect their femoral artery blood and elbow venous blood after angiography. Another eighteen of the healthy volunteers have signed the agreements to only collect their elbow venous blood after angiography.

### Ethics Statement

This study was approved by the Human Research Ethics Committee of Xinhua Hospital affiliated with Shanghai Jiaotong University School of Medicine. Each participant signed a written informed consent form.

### Cell Isolation

Blood mononuclear cells were isolated from the samples of the femoral artery and peripheral blood of LEASO patients and HCs. Whole blood containing an acid citrate dextrose anticoagulant (Biological Specialty Corporation, Colmar, PA, USA) was carefully layered over an equal volume of Ficoll (Shanghai, China; ρ=1.077 ± 0.002 g/ml) density gradient medium. Tubes containing the blood and Ficoll were centrifuged for 20 min at 2,000 rpm. Isolated arterial blood mononuclear cells (ABMCs) and peripheral blood mononuclear cells (PBMCs) were washed two times in phosphate-buffered saline (PBS) and centrifuged for 10 min at 1,200 rpm and cultured in RPMI 1640 supplemented with 10% heat-inactivated fetal bovine serum (FBS; Gibco, Grand Island, NY, USA), 100 U/ml penicillin, 100 μg/ml streptomycin, and 1 μg/ml amphotericin B at 37°C in 95% air and 5% CO_2_. CD4^+^ T cells were isolated by magnetic affinity cell sorting using CD4 microbeads (Miltenyi Biotec, Auburn, CA, USA) with LS columns following the manufacturer’s instructions.

### Cell Treatment

CD4^+^T cells from HCs were stimulated with different concentrations of ox-LDL (0, 1, 10, 50, and 100 µg/ml) for 48 h. Some CD4^+^T cells were also cultured with a plate-bound anti-CD3 antibody (OKT-3; 5 μg/ml) plus anti-CD28 antibody (28.2; 1 μg/ml) to get activated.

For Tim-3- and PD-1-targeting experiments, arterial CD4^+^T cells were cultured (5 × 10^5^ per well) in the presence of 10 μg/ml anti-Tim-3 (clone F38-2E2; BioLegend San Diego, CA, USA) and anti-PD-1 (clone EH12.2H7, BioLegend San Diego, CA, USA), or isotype control. After 48 h, the culture supernatants were collected and further analyzed by flow cytometry (FCM).

For intracellular cytokine analysis, brefeldin A (a Golgi inhibitor) (10 mg/ml, Biolegend, San Diego, CA, USA) was used for 4 h (at the end of the culture) to block the secretion of cytokines into the media after the activation of cells by phorbol 12-myrstate 13-acetate (PMA) (50 ng/ml, 12 h) and ionomycin (1 μg/ml, 12 h). Cells were harvested and analyzed by FCM for intracellular cytokine production.

### Flow Cytometry

Cell surface molecular expression and intracellular cytokine production were evaluated using FCM. Fluorescein isothiocyanate (FITC)-conjugated anti-human CD4, PD-1, IL-17A, or CD127, Alexa Fluor 488-conjugated anti-human IL-4, PE-conjugated anti-human CD4, Tim-3 or GATA-3, PE/CY7-conjugated anti-human CD4, CD69, CD45RO, IL-10, TNF-α, IFN-γ or TGF-β1, APC-conjugated anti-human PD-1, CTLA-4, IL-4 or IFN-γ, Brilliant Violet 421-conjugated anti-human CD127, HLA-DR, IL-17A, IL-4, IL-6, T-bet, IFN-γ or Ki67, and Brilliant Violet 510-conjugated anti-human CD4 (Biolegend, USA) were used. For intracellular staining, cells were fixed and permeabilized using a Fix/Perm kit (Biolegend, USA). FCM analysis was performed on a Beckman–Coulter CyAN ADP flow cytometer and analyzed with FlowJo software (Tree Star, Ashland, OR, USA).

### Statistical Analysis

One-way analysis of variance (ANOVA) was used to evaluate differences. A p- value of less than 0.05 was considered statistically significant. For variables with a p- value of less than 0.05 in ANOVA, the *post-hoc* Dunnett t-test was performed to determine the differences between each group. All analyses were carried out using the GraphPad Prism 7 software (GraphPad, San Diego, CA, USA).

## Results

### Higher Proportion of Tim-3^+^PD-1^+^CD4^+^T Cells in LEASO

**Figure 1:**
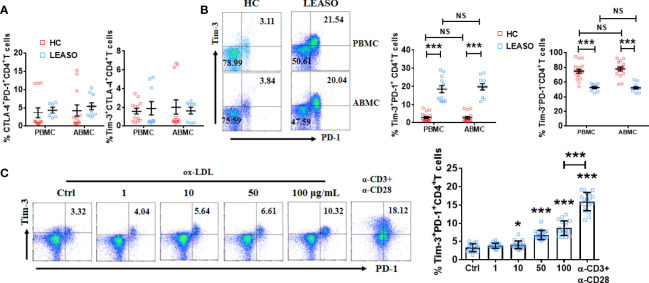
Higher proportion of Tim-3^+^PD-1^+^CD4^+^T cells in LEASO. **(A)** Frequency of CTLA-4 and PD-1 co-expression and Tim-3 and CTLA-4 co-expression on CD4^+^T cells from the femoral artery blood of the affected limb [arterial blood mononuclear cells (ABMCs)] and peripheral venous blood (peripheral blood mononuclear cells (PBMCs)] of LEASO patients and HCs, n=6-12. **(B)** The percentage of Tim-3^+^PD-1^+^CD4^+^T cells and Tim-3^-^PD-1^-^CD4^+^T cells in the circulation of LEASO patients and HCs, n=10–18. **(C)** Quantification of flow cytometric analysis of Tim-3 and PD-1 co-expression on peripheral CD4^+^T cells of HCs treated with the indicated concentrations of ox-LDL for 48 h. The α-CD3 and/or α-CD28 antibodies were used in some wells. n=18, Data represent mean ± standard error of the mean (SEM). *P < 0.05, ***P < 0.001, NS, no significance, P>0.05.

We first compared the pairwise co-expression of Tim-3, PD-1, and CTLA-4 on arterial and peripheral lymphocytes in LEASO patients (stage III or IV according to the Fontaine classification system) and HCs by FCM. As shown in **Figure 1A**, the frequency of CTLA-4^+^PD-1^+^CD4^+^T cells and Tim-3^+^CTLA-4^+^CD4^+^T cells was similar in LEASO patients and HCs both in arterial and peripheral lymphocytes. While the Tim-3 and PD-1 co-expression was elevated on CD4^+^T cells in the femoral artery blood of the affected limb and peripheral venous blood of LEASO patients than that in HCs. We also compared Tim-3 and PD-1 expression on arterial and peripheral blood CD4^+^T cells from the same donors and found that the co-expression levels were accordant in blood from the femoral artery of the affected limb and peripheral vein (**Figure 1B**).

Then, why is Tim-3 and PD-1 co-expression on CD4^+^T cells higher in LEASO? It is generally suggested that ox-LDL induces endothelial injury, activates CD4^+^T cells, and further spurs the initiation and progression of LEASO (5, 18). We stimulated peripheral CD4^+^T cells from HCs with different concentrations of ox-LDL *in vitro* and found that ox-LDL upregulated Tim-3 and PD-1 co-expression in a dose-dependent manner (**Figure 1C**). As activation with an anti-CD3/CD28 antibody was also sufficient to increase Tim-3 and PD-1 co-expression on CD4^+^T cells *in vitro* (**Figure 1C**), we speculated that ox-LDL contributed to the higher proportion of Tim-3^+^PD-1^+^CD4^+^T cells in LEASO by activating CD4^+^T cells.

### CD4^+^T Cells Co-Expressing Tim-3 and PD-1 From LEASO Patients Displayed a Distinctive Phenotype Compared to Tim-3^-^PD-1^-^CD4^+^T Cells *In Vitro*

**Figure 2:**
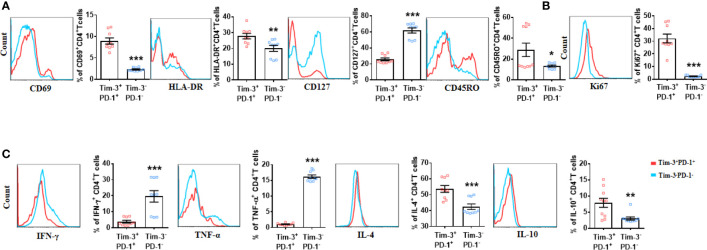
CD4^+^T cells co-expressing Tim-3 and PD-1 from LEASO patients displayed a distinctive phenotype compared to Tim-3^-^PD-1^-^CD4^+^T cells *in vitro*. **(A)** ABMCs from the LEASO (n = 9) were stained with antibodies against CD4, CD69, HLA-DR, CD127, and CD45RO and then analyzed by flow cytometry. **(B)** Quantification of Ki67 staining in Tim-3^+^PD-1^+^CD4^+^T cells and Tim-3^-^PD-1^-^CD4^+^T cells from LEASO, n = 9. **(C)** Cytokine production by Tim-3^+^PD-1^+^CD4^+^T cells and Tim-3^-^PD-1^-^CD4^+^T cells from LEASO, n = 9. Data represent mean ± SEM. *P < 0.05, ** P < 0.01, *** P < 0.001.

Given the increased co-expression of Tim-3 and PD-1, Tim-3^+^PD-1^+^CD4^+^T cells during human LEASO could be dysfunctional. Alternatively, the expression of inhibitory receptors could also be the consequence of the local activation of CD4^+^T cells. To study these two possibilities, we first examined the activation extent of Tim-3^+^PD-1^+^ and Tim-3^-^PD-1^-^CD4^+^T cells by using the activation and memory markers. This investigation showed that Tim-3^+^PD-1^+^CD4^+^T cells expressed higher levels of activation (CD69 and HLA-DR) and memory markers (CD45RO) but lower CD127 (as CD4^+^T cells are activated, they lose the expression of CD127) than Tim-3^–^ PD-1^–^CD4^+^T cells (**Figure 2A**). As seen in **Figure 2B**, the majority of the expanded population (assessed using the Ki67^+^ cells) of CD4^+^T cells was Tim-3^+^PD-1^+^, while the proliferation of Tim-3^-^PD-1^-^CD4^+^ T cells was restricted in LEASO.

Next, we stimulated fresh CD4^+^T cells from the femoral artery blood of the affected limb with PMA and ionomycin to assess effector cytokine production *in vitro*. After exposure to PMA and ionomycin for 4 h, the percentage of IFN-γ- and TNF-α- producing cells in Tim-3^+^PD-1^+^CD4^+^T cells were reduced compared with Tim-3^-^PD-1^-^CD4^+^T cells; the expression of IL-4 and IL-10 was significantly increased by Tim-3^+^PD-1^+^CD4^+^T cells (**Figure 2C**). Meanwhile, the TGF-β1, IL-17A and IL-6 expression between the two groups had no difference (**Figure S1**). Together, at least, these data indicated that the CD4^+^T cells that co-expressed Tim-3 and PD-1 were not exhausted in LEASO.

### Altered Phenotype of Tim-3^+^PD-1^+^CD4^+^T Cells From LEASO Patients Compared to Those From Healthy Controls *In Vitro*

**Figure 3:**
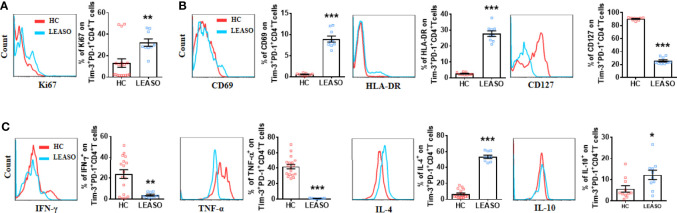
Altered phenotype of Tim-3^+^PD-1^+^CD4^+^T cells from LEASO patients compared to those from HCs *in vitro*. **(A)** Expression of Ki67 by Tim-3^+^PD-1^+^CD4^+^T cells from HC (n = 18) and LEASO (n = 9). **(B)** Expression of activation markers by Tim-3^+^PD-1^+^CD4^+^T cells from HCs (n = 9) and LEASO (n = 9). **(C)** Flow cytometric analysis and the quantification of flow cytometric analysis of pro-atherogenic cytokines (IFN-γ and TNF-α) and anti-atherogenic cytokines (IL-4 and IL-10) in Tim-3^+^PD-1^+^CD4^+^T cells from HCs (n = 11-18) and LEASO (n = 9). Data represent mean ± SEM. *P < 0.05, **P < 0.01, ***P < 0.001.

As the number of Tim-3^+^PD-1^+^CD4^+^T cells was much higher in LEASO, and Tim-3^+^PD-1^+^CD4^+^T cells were associated with anti-atherogenic cytokine production, we further explored the possible clinical significance of Tim-3 and PD-1 by analyzing samples from HCs and patients who experienced LEASO *in vitro*. Apart from the increased frequency, the expression of Ki67 (**Figure 3A**), CD69, and HLA-DR (**Figure 3B**) on Tim-3^+^PD-1^+^CD4^+^T cells was also increased in LEASO compared to those in HCs. Additionally, consistent with the more activated phenotype of Tim-3^+^PD-1^+^CD4^+^T cells in LEASO, the expression of CD127 (**Figure 3B**) on Tim-3^+^PD-1^+^CD4^+^T cells was decreased in LEASO. Furthermore, compared to HCs, Tim-3^+^PD-1^+^CD4^+^T cells produced lower TNF-α and IFN-γ but more IL-4 and IL-10 in LEASO patients (**Figure 3C**). Meanwhile, we could not detect the difference of the memory marker CD45RO (**Figure S2A**), IL-17A, and TGF-β1 (**Figure S2B**) expression on Tim-3^+^PD-1^+^CD4^+^T cells between HCs and LEASO.

### Effects of *In Vitro* Targeting Tim-3 and PD-1 Signaling Pathways on CD4^+^ T Cells From LEASO Patients

**Figure 4:**
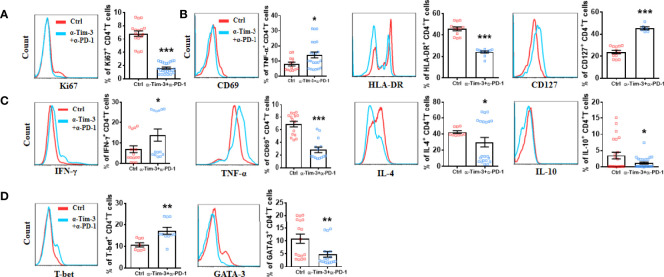
Effect of *in vitro* targeting Tim-3 and PD-1 signaling pathways on CD4^+^ T cell function from LEASO patients. Quantification of flow cytometric analysis of Ki67 expression **(A)**, activation markers **(B)**, cytokine production **(C)**, and Th1-type and Th2-type transcription factors **(D)** by CD4^+^ T cells from LEASO cultured for 48 h in the presence or absence anti-Tim-3 and anti-PD-1 antibodies. Data represent mean ± SEM (n = 16). *P < 0.05, **P < 0.01, ***P < 0.001, compared with the control group.

Next, we utilized anti-Tim-3 and anti-PD-1 antibodies to inhibit the Tim-3 and PD-1 pathways to further explore the relationship between Tim-3 and PD-1 co-expression and CD4^+^ T-cell function in LEASO. We stimulated CD4^+^T cells from LEASO patients with anti-CD3/CD28 in the presence or absence of antibodies blocking Tim-3/PD-1 pathways *in vitro*. After 48 h, the expression levels of surface molecules and intracellular cytokines on CD4^+^T cells were analyzed. Unlike the results observed in infections and cancer (19), the combinations of blocking Tim-3 and PD-1 resulted in a profound reduction in Ki67 expression (**Figure 4A**) and the activation (**Figure 4B**) of CD4^+^T cells in LEASO. Co-treatment with anti-Tim-3 and anti-PD-1 antibodies notably reduced the ability of CD4^+^T cells to produce IL-4 and IL-10 but increased the production of Th1-type TNF-α and IFN-γ (**Figure 4C**). We then examined the expression of the master transcription factors associated with Th1 and Th2 cells, namely, T-bet and GATA-3 (20), respectively. Consistent with the results identified in **Figure 4C**, the expression of T-bet was upregulated, while GATA-3 on CD4^+^T cells was downregulated following a combined antibody blockade (**Figure 4D**).

## Discussion

In the present *in vitro* study, we confirmed the higher co-expression of Tim-3 and PD-1 on CD4^+^ T cells in higher-stage LEASO patients than that from HCs both within the femoral artery blood of the affected limb and peripheral venous lymphocytes, while Tim-3^-^PD-1^-^CD4^+^T cells were less abundant in LEASO. As LEASO is a manifestation of systemic atherosclerosis in the limbs, we speculated that increased Tim-3 and PD-1 expression might also be observed in atherosclerosis and atherosclerosis-related diseases. Whether a higher co-expression of Tim-3 and PD-1 on CD4^+^ T cells is specially related to LEASO remains to be determined. We previously reported the slight increase (no significant difference) in the Tim-3^+^PD-1^+^CD4^+^T cell proportion in lower stage (stage II or III according to the Fontaine classification system) LEASO patients (21). These results, at least, indicated that Tim-3 and PD-1 might be promising an early warning of the higher-stage progression of LEASO. Notably, the co-expression of Tim-3 and PD-1 was associated with an active phenotype and increased anti-atherogenic cytokine production but decreased pro-atherogenic cytokine production by CD4^+^T cells from the femoral artery blood of the affected limb. We further showed that targeting Tim-3 and PD-1 signaling pathways in CD4^+^T cells resulted in a decrease in anti-atherogenic cytokine production and in an augmentation in the generation of pro-atherogenic cytokines. These findings, for the first time, highlight the important roles that Tim-3 and PD-1 signaling pathways may play in the regulation of CD4^+^ T cell function in LEASO.

Previous studies have revealed that single PD-1, Tim-3, or CTLA-4 pathway signaling has anti-atherogenic effects during the progress of murine atherosclerosis (13–15). However, the co-expression of all these inhibitory receptors on CD4^+^T cells in patients with LEASO has not been studied before. Here, we demonstrated that CD4^+^T cells in LEASO had a significantly higher co-expression of Tim-3 and PD-1. On the other hand, we did not find any difference in the frequency of CTLA-4^+^PD-1^+^CD4^+^T cells and Tim-3^+^CTLA-4^+^CD4^+^T cells in LEASO patients and HCs. The selectively increased expression of Tim-3 and PD-1 on CD4^+^T cells in LEASO suggests that they may be involved in the regulation of antigen-specific responsiveness of T cells in LEASO. Ox-LDL is widely considered as the main antigen involved in the inflammation of LEASO (5, 18), and ox-LDL-specific CD4^+^T cells have been suggested to play a crucial role in atherosclerosis (22). We further found that ox-LDL contributed to the higher proportion of Tim-3^+^PD-1^+^CD4^+^T cells in LEASO by activating CD4^+^T cells in a dose-dependent manner.

Given that the expression of individual inhibitory receptor on T cells can be induced by T cell activation (23), but the expression of increasing numbers of inhibitory receptors often coincides with the gradual loss of T cell functions (19), we invested the activation status and cytokine production profile of CD4^+^T cells from LEASO in relation to the expression of Tim-3 and PD-1. Interestingly, Tim-3^+^PD-1^+^CD4^+^T cells displayed a more active status and produced more IL-4 and IL-10. However, they displayed reduced IFN-γ and TNF-α and comparable TGF-β1, IL-17A, and IL-6 compared to Tim-3^-^PD-1^-^CD4^+^T cells. In addition, compared to HCs, this Tim-3^+^PD-1^+^CD4^+^T cell subset in LEASO expressed higher Ki67, IL-4, and IL-10 but lower IFN-γ and TNF-α and displayed a more active status. These findings indicated that the CD4^+^T cells that express Tim-3 and PD-1 are probably ox-LDL-reactive T cells, which may continue to upregulate the expression of Tim-3 and PD-1 in response to chronic stimulation by ox-LDL to prevent further inflammation in LEASO.

The pro-inflammatory and pro-atherogenic nature of Th1-type cytokines IFN-γ and TNF-α and the anti-inflammatory and anti-atherogenic nature of IL-10 is well documented (11). However, whether Th2 cells are pro-atherogenic or anti-atherogenic remains unclear. IL-4 was shown to antagonize Th1 responses and diminish atherogenic lesion formation in *Apoe^-/-^* mice (24), whereas another study has shown that the depletion of IL-4 is anti-atherogenic in the *Ldlr^-/-^* mice fed with a high-fat diet (25). However, mouse models may not entirely represent the physiology of human diseases, as individuals with a high number of Th2 cells in the PBMC fraction have a lower subclinical atherosclerosis burden, as measured by common carotid intimal media thickness, than individuals with low Th2 cell counts (26). Given the anti-inflammatory and Th1-inhibitory potency of IL-4, we speculated that the Tim-3^+^PD-1^+^CD4^+^T cell subset in LEASO expressed higher IL-4 and IL-10, but lower IFN-γ and TNF-α are anti-atherogenic.

Not surprisingly, the impact of blocking Tim-3 and PD-1 on the function of CD4^+^T cells in LEASO agreed well with the observed alteration of their phenotype between Tim-3^+^PD-1^+^CD4^+^T cells and Tim-3^-^PD-1^-^CD4^+^T cells. The blockade of Tim-3 and PD-1 pathways can suppress the proliferation and activation of CD4^+^T cells and inhibit the Th2 bias but enhance the expression of IFN-γ, TNF-α, and T-bet on CD4^+^T cells. Immune checkpoint inhibitors are considered the standard of care for several cancers and chronic infections (16). Immune checkpoint inhibitors are monoclonal antibodies directed against co-inhibitory molecules, in particular, CTLA-4, PD-1, and Tim-3. Nowadays, combination therapies (such as combining anti-Tim-3 and anti-PD-1) were thought to be a promising novel therapeutic approach for several types of human malignancies (27). As atherosclerosis develops gradually over years or decades and immune checkpoint inhibitors have been implemented in the clinic only in the past decade, whether the patients who are treated with immune checkpoint inhibitors have an increased risk of developing atherosclerotic diseases is currently unknown (28). However, combined anti-PD-1 and anti-CTLA-4 signaling therapy increased the T cell/macrophage ratio in coronary atherosclerotic plaques, which reflects a strong T cell-driven inflammation that is associated with plaque instability (29). In our study, we also found that the combination blockade of Tim-3 and PD-1 induced the dysfunction of CD4^+^T cells in LEASO. Importantly, the cardiovascular safety must be a criterion considered in modulating the dose and function of immune checkpoint inhibitors to reverse T cell exhaustion in tumors.

**Figure 5:**
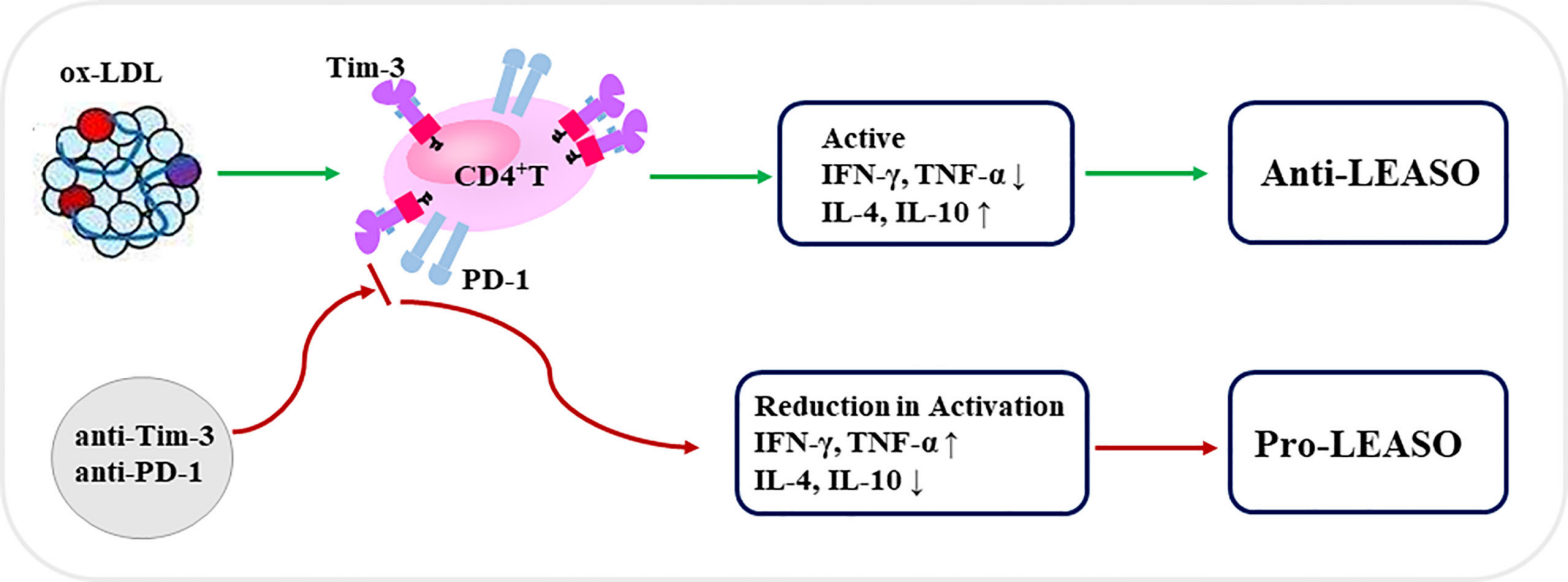
Schematic diagram of Tim-3 and PD-1 signals in regulating CD4^+^T cell function during LEASO. Ox-LDL increased the co-expression of Tim-3 and PD-1 on CD4^+^T cells during LEASO. Tim-3/PD-1 pathways, in turn, may operate within the functional immune-modulatory network to prevent further inflammation in LEASO as Tim-3^+^PD-1^+^CD4^+^T cells displayed a more active status and produced more anti-atherogenic cytokines. Meanwhile, the blockade of Tim-3 and PD-1 signaling pathways aggravated the pro-inflammatory Th1 responses predominating in LEASO, which might be conducive to the development of LEASO.

In summary (**Figure 5**), our *in vitro* data demonstrate, for the first time, that the co-expression of Tim-3 and PD-1 on CD4^+^T cells is upregulated in human higher stage LEASO, and ox-LDL contributed to the higher proportion of Tim-3^+^PD-1^+^CD4^+^T cells by activating CD4^+^T cells in a dose-dependent manner. These results indicated that Tim-3 and PD-1 might be promising an early warning of a higher-stage progression of LEASO. Tim-3^+^PD-1^+^CD4^+^T cells from the femoral artery blood of the affected limb displayed a more active status and produced more anti-atherogenic cytokines, and the blockade of Tim-3 and PD-1 signaling pathways aggravated the pro-inflammatory Th1 responses predominant in LEASO. The blockade of Tim-3 and PD-1 to improve immune cell responses is considered as a novel strategy for the treatment of tumors and chronic infections. However, with the risk of developing atherosclerotic cardiovascular diseases (especially LEASO), cardiovascular safety would ultimately be an individualized decision made with a careful consideration of potential benefits and risks.

## Data Availability Statement

The original contributions presented in the study are included in the article/supplementary material. Further inquiries can be directed to the corresponding authors.

## Ethics Statement

This study was approved by the Ethics Committee of Xinhua Hospital Affiliated to Shanghai Jiaotong University School of Medicine. The patients/participants provided their written informed consent to participate in this study.

## Author Contributions

SW and MQ conceived experiments. SW, LYC, MQ, and LTC carried out experiments and analyzed data. MQ, LYC, YD, and JO coordinated the sample collection, data interpretation, literature search, and figure preparation. SW drafted the manuscript. MQ and LYC revised the manuscript. All authors reviewed the manuscript.

## Funding

This work was supported by grant from the Nature Science Foundation of Shanghai (21ZR1410500 to SW), the Shanghai Chenguang Program (18CG09 to SW), the Natural Science Foundation of Tibet Autonomous Region (XZ202101ZR0010G and XZ2020ZR-ZY41 (Z) to MQ), the Medical transformation cross fund of Shanghai Jiaotong University (ZH2018QNA60 to MQ), and the Shanghai Sailing Program (19YF1404100 to LYC).

## Conflict of Interest

The authors declare that the research was conducted in the absence of any commercial or financial relationships that could be construed as a potential conflict of interest.

## Publisher’s Note

All claims expressed in this article are solely those of the authors and do not necessarily represent those of their affiliated organizations, or those of the publisher, the editors and the reviewers. Any product that may be evaluated in this article, or claim that may be made by its manufacturer, is not guaranteed or endorsed by the publisher.

## Supplementary Material

Supplementary Figure 1 Cytokine production in Tim-3^+^PD-1^+^CD4^+^ T and Tim-3^-^PD-1^-^CD4^+^T cells during LEASO. Quantification of flow cytometric analysis of TGF-β1, IL-17A and IL-6 on Tim-3^+^PD-1^+^CD4^+^T cells and Tim-3^-^PD-1^-^CD4^+^T cells from LEASO. Data represent mean ± SEM.

Supplementary Figure 2 Phenotype of Tim-3^+^PD-1^+^CD4^+^ T cells in HCs and LEASO. Quantification of flow cytometric analysis of CD45RO **(A)** and TGF-β1 and IL-17A **(B)** by Tim-3^+^PD-1^+^CD4^+^T cells from HCs and LEASO. Data represent mean ± SEM.
