# Supplementary figures and images for: Increased Level of Tim-3+PD-1+CD4+T Cells With Altered Function Might Be Associated With Lower Extremity Arteriosclerosis Obliterans
Source: Front Immunol. 2022 Jun 10;13:871362. doi: 10.3389/fimmu.2022.871362 (PMC9229777; doi:10.3389/fimmu.2022.871362)

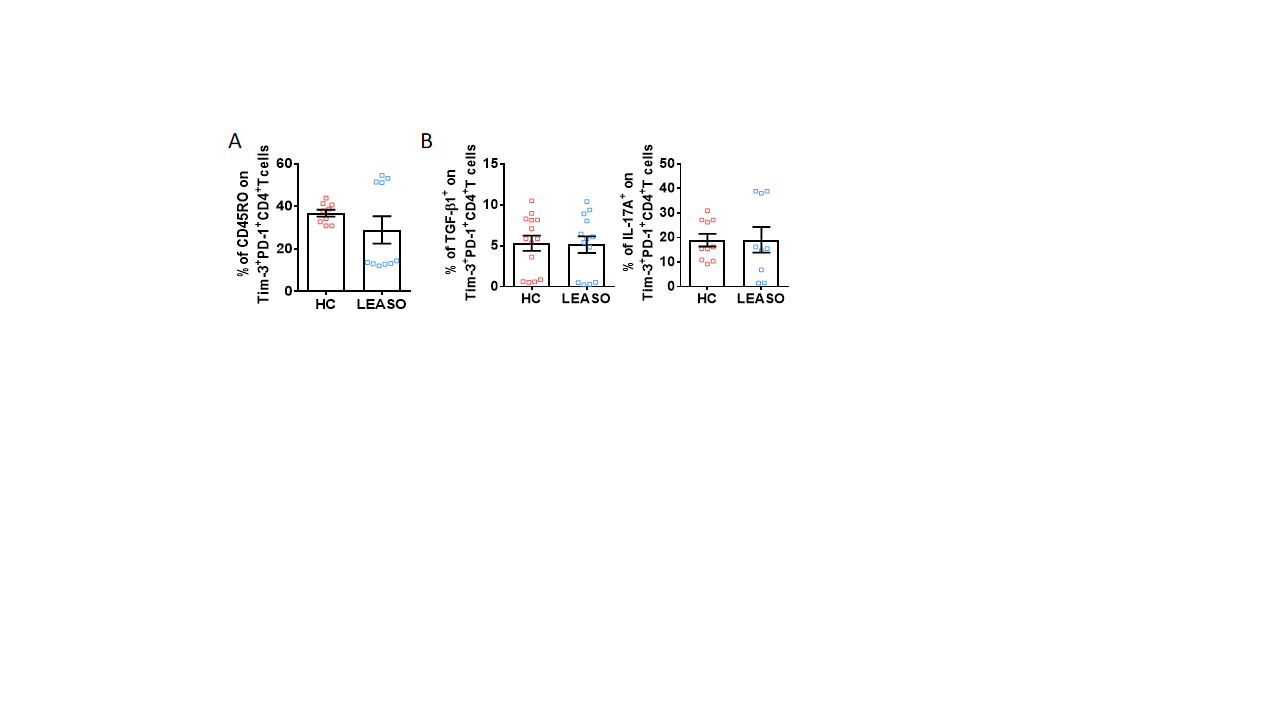

Supplement: Supplementary Figure 1 — Cytokine production in Tim-3+PD-1+CD4+ T and Tim-3-PD-1-CD4+T cells during LEASO. Quantification of flow cytometric analysis of TGF-β1, IL-17A and IL-6 on Tim-3+PD-1+CD4+T cells and Tim-3-PD-1-CD4+T cells from LEASO. Data represent mean ± SEM. [file Image_1.tif]

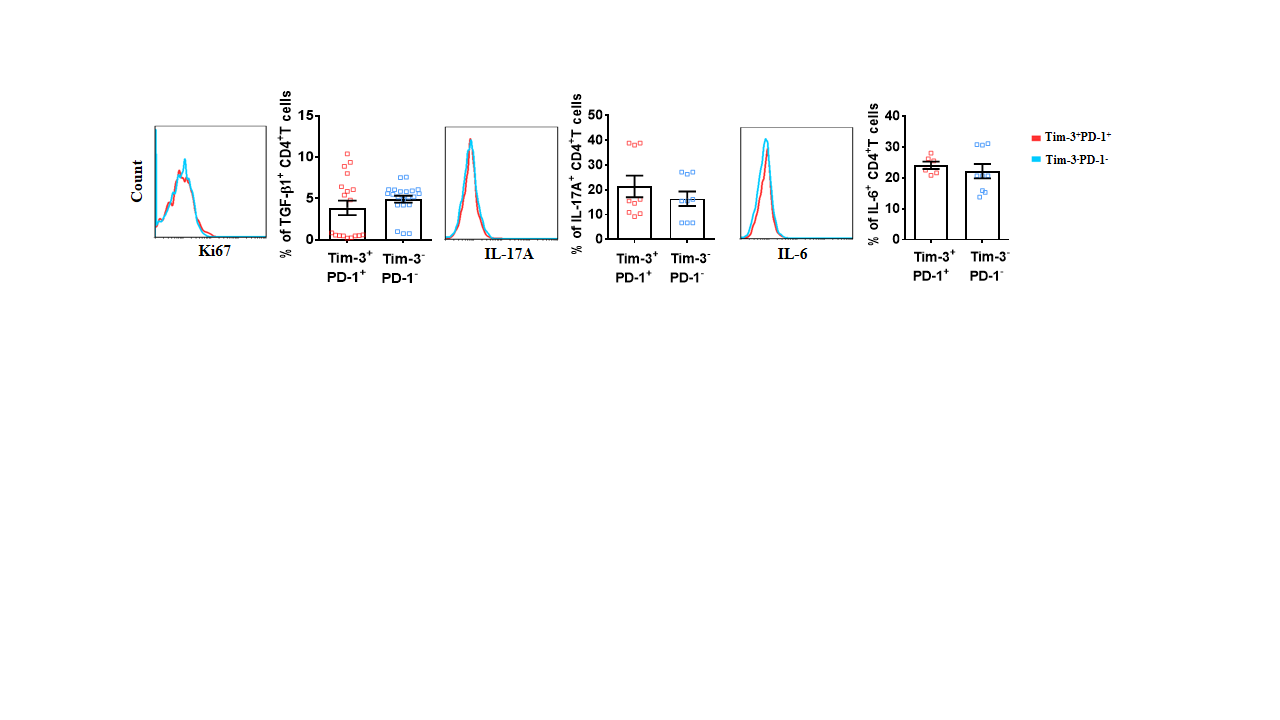

Supplement: Supplementary Figure 2 — Phenotype of Tim-3+PD-1+CD4+ T cells in HCs and LEASO. Quantification of flow cytometric analysis of CD45RO (A) and TGF-β1 and IL-17A (B) by Tim-3+PD-1+CD4+T cells from HCs and LEASO. Data represent mean ± SEM. [file Image_2.tif]
